# Radiological characteristics of screen-detected lung cancers: predictive for histological subtype?

**DOI:** 10.1186/1470-7330-14-S1-P20

**Published:** 2014-10-09

**Authors:** MA Heuvelmans, E Salters, HJM Groen, P De Jong, WPTM Mali, M Oudkerk, R Vliegenthart

**Affiliations:** 1 Center for Medical Imaging – North East Netherlands, Department of Radiology, University of Groningen, University Medical Center Groningen, Groningen, Netherlands; 2 Department of Pulmonology, University of Groningen, University Medical Center Groningen, Groningen, Netherlands; 3 Department of Radiology, University Medical Center Utrecht, Utrecht, Netherlands

## Aim

To evaluate CT-morphological features of lung cancers detected in a CT lung cancer screening trial, and to determine the correlation between CT-morphological features and the histopathological diagnosis of screen-detected cancers.

## Methods

197 solid lung cancers (192 participants) detected in all four screening rounds of the Dutch-Belgian randomized lung cancer screening trial (NELSON) were included. CT-morphological features included nodule shape, margin, location, volume, and volume-doubling time (VDT). Based on histopathology, cancers were divided into four groups: adenocarcinoma (N=114), squamous cell carcinoma (N=37), large cell carcinoma (N=28) and neuro-endocrine cancers (N=18). Data were analyzed using ANOVA, Chi-square and Fischer’s exact test.

## Results

Mean participant age was 61.3 years (95%-confidence interval [CI]: 60.5-62.2), and 160/192 (83.3%) were male. In all four histopathologic groups, the majority of cancers had a spherical nodule shape (70.6-95.8%). Margins of malignant nodules were most often lobulated (39.3-45.9%) and spiculated (22.2-35.7%), without statistically significant difference between histopathological groups. Most cancers (63.5%) were located in the upper lobes, adenocarcinomas significantly more often (71,1%) than other types of cancers (p=0.004). Adenocarcinomas had a higher mean VDT than large cell carcinomas (214.8 days, 95%-CI: 186.2-243.4 days vs 96.8 days, 95%-CI: 15.8-177.9 days [p<0.05]). VDTs of other histopathological subgroups did not differ significantly.

## Conclusion

Only VDT and location in the upper versus lower lobes are associated with histopathological diagnosis of screen-detected lung cancers. No discrimination can be made between different histopathologic cancer types based on CT-morphological features alone.

